# Association between copper and Achilles tendon disease: a two-sample Mendelian randomization study

**DOI:** 10.3389/fnut.2024.1505636

**Published:** 2024-11-13

**Authors:** TianYang Chen, Yan Gu, ZiHao Zhang, ZhaoLiang Chen, JingQuan Zhang, Xiangyang Leng

**Affiliations:** Changchun University of Chinese Medicine, Changchun, China

**Keywords:** Achilles tendon disease, Mendelian randomization analysis, copper, micronutrients, causal research

## Abstract

**Background:**

There is a clear association between micronutrients and Achilles tendon disease (AT). An increase in micronutrients may alleviate AT symptoms and have a therapeutic effect. The aim of this study is to clarify the causal relationship between 15 micronutrients (copper, zinc, magnesium, vitamins A, C, E, D, B6, B12, folic acid, carotene, iron, selenium, calcium, and potassium) and AT.

**Methods:**

We employed the Mendelian randomization (MR) method to analyze the causal effects of micronutrients on the risk of AT. The SNPs related to micronutrients were obtained from a large-scale genome-wide association study (GWAS) of circulating micronutrients in European populations. Outcome data were obtained from a meta-analysis of AT in European-ancestry participants from the Finnish FINNGEN BIOBANK. The main analysis was conducted using the inverse variance weighting (IVW) method, with additional sensitivity and pleiotropy analyses performed.

**Results:**

Inverse variance weighting results indicated a causal relationship between copper and AT (*P* = 0.003, OR = 0.899, 95% CI = 0.839–0.964). Sensitivity analysis validated the robustness and reliability of this finding.

**Conclusion:**

This study revealed a causal relationship between copper and AT, with copper serving as a protective factor. This provides evidence of the causality between copper and AT, offering new insights for clinical research and therapeutic approaches in AT.

## 1 Introduction

The Achilles tendon is the strongest tendon in the human body and plays a vital role in foot movement (1). Achilles tendon disease (AT) essentially represents a failure of the tendon healing response, where under conditions of ischemia, high temperature, and hypoxia, tendon cells in the Achilles tendon undergo degeneration, collagen fibers are damaged, and non-collagenous matrix increases (2), Tendon cell apoptosis and free radical damage following injury are also associated with AT (3–5). Ischemia and hypoxia in the Achilles tendon promote new blood vessel formation; however, these vessels are highly permeable and fail to effectively perfuse the tissue, making healing difficult. Moreover, these new blood vessels may be accompanied by the formation of small nerves, which are associated with localized pain (6, 7). Evidence suggests that aging leads to abnormal changes in the expression of various genes and the production of different types of matrix proteins in tendons, potentially resulting in tendon degeneration, aging, and impaired healing (2). These factors may reduce the Achilles tendon’s resistance to stress and strain, negatively affecting its ability to transmit force and generate power (7). The natural course and clinical progression of AT are still unclear. Symptoms of AT include pain, swelling, and dysfunction in or around the Achilles tendon, significantly affecting patients’ quality of life (8, 9). Some studies indicate a high incidence of this disease among middle-aged men and sports enthusiasts (10). However, further in-depth research is needed on the treatment and prevention of AT.

Micronutrients play a crucial role in maintaining overall health and are closely linked to AT (AT) (11, 12). Vitamin C is crucial for the enzymatic synthesis of collagen and various proteoglycans found in tendons (13). Additionally, vitamin C acts as a transcriptional promoter of collagen synthesis. An animal study reported that high-dose oral vitamin C supplementation can significantly accelerate the healing of Achilles tendon rupture (14). Another animal study found that a vitamin D-deficient diet results in delayed healing of shoulder tendons and decreased biomechanical strength (15). 60% of the human body’s zinc content is located in muscles, and 30% is in the bones (16). Zinc enhances the body’s natural defense mechanisms and mitigates oxidative stress by preventing the formation of free reactive oxygen species (ROS) through copper-zinc superoxide dismutase in the cytosol and mitochondrial membranes, as well as the less active copper-zinc superoxide dismutase in plasma and biological fluids (17). Vitamin B12 can effectively alleviate pain caused by partial transection injuries of the Achilles tendon, inhibit ectopic nerve sprouting, and accelerate tendon repair by suppressing protease-activated receptor 2 (PAR2) activation in neurons (18). Cyclic strain in tendon cells activates stress-activated protein kinase (SAPK/JNK) through calcium-mediated signaling, with this stress response being sensitive to both the frequency and magnitude of strain. Cyclic strain promotes tendon health (19). Ferroptosis can be mitigated by reducing lipid peroxidation and iron accumulation in tendon cells, which alleviates collagenase-induced tendinopathy (20).

The MR analysis is an “natural experiment” epidemiological method that uses genetic variation as an instrumental variable to strengthen the random inference of causal effects on modifiable exposures (risk factors) (21–23). In MR, single nucleotide polymorphisms (SNPs) associated with exposure events are used as instrumental variables (IVs). Genetic variation is randomly assigned at conception, and since the instrumental variable is independent of other confounders, MR can assess the causal relationship between previously observed exposures and outcomes, effectively avoiding confounding bias present in traditional epidemiological studies (24). In this study, we used the MR method to explore the relationship between micronutrients and AT, aiming to better understand the role of micronutrients in AT and to inform clinical diagnosis and treatment.

## 2 Materials and methods

### 2.1 Study design

**Figure 1:**
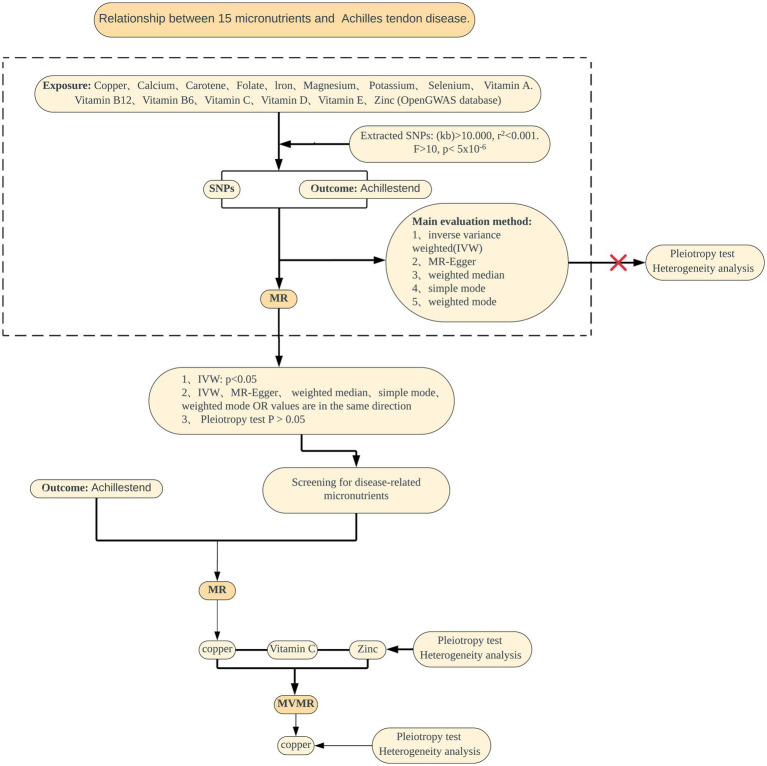
Summary of the MR study design for the relationship between 15 micronutrients and AT. MR, Mendelian randomization; SNPs, single nucleotide polymorphisms; IVW, inverse variance weighted; MVMR, Multivariable Mendelian randomization.

**Figure 2:**
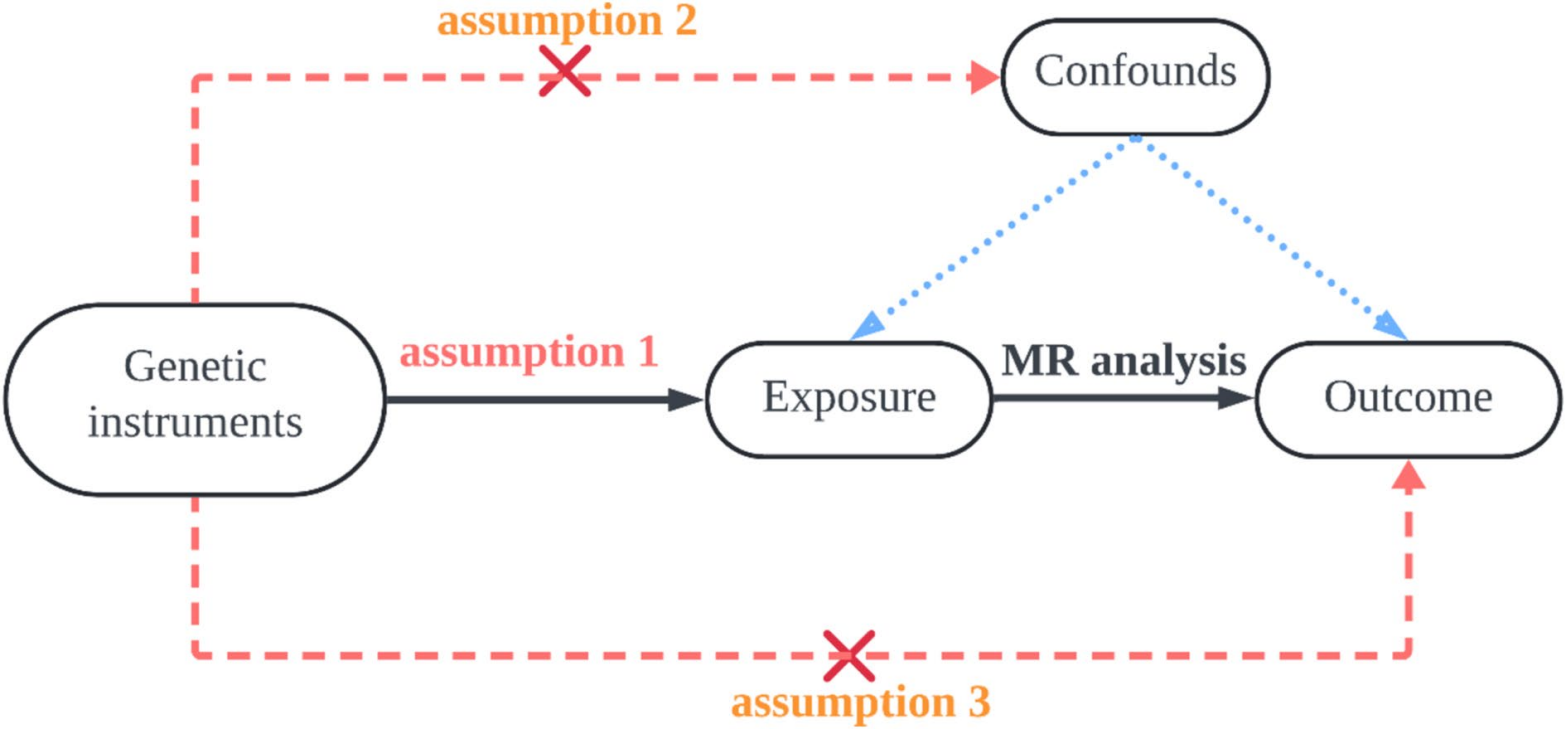
The first hypothesis is that the selected SNP is significantly associated with the exposure factor (micronutrient); the second hypothesis is that the SNP must be independent of potential confounders between exposure and outcome; the third hypothesis is that the SNPs are not directly related to Achilles tendinopathy, but only causally linked through micronutrients.

The design is illustrated in Figure 1. Data for the 15 exposures were sourced from the published Open GWAS public database. GWAS data for one outcome were derived from the FinnGen Biobank. Both the exposure and outcome data were derived from participants of European ancestry. This MR analysis adheres to three fundamental assumptions, as shown in Figure 2. The first assumption is that the chosen SNPs are significantly associated with the exposure factors (micronutrients). The second assumption is that the SNPs must be independent of confounding factors between the exposure and the outcome. The third assumption is that the SNPs are not directly associated with AT and can only be causally linked through micronutrients.

### 2.2 Data sources

In this study, the genome-wide association study (GWAS) data for micronutrients were sourced from IEU OpenGWAS,^1^ and the summary statistics for 15 micronutrients are shown in Table 1. First, the OpenGWAS data for copper included 2,603 Europeans, with GWAS ID ieu-a-1073. The OpenGWAS data for calcium included 64,979 Europeans, with GWAS ID ukb-b-8951. The OpenGWAS data for carotene included 64,979 Europeans, with GWAS ID ukb-b-16202. The OpenGWAS data for folate included 64,979 Europeans, with GWAS ID ukb-b-11349. The OpenGWAS data for iron included 64,979 Europeans, with GWAS ID ukb-b-20447. The OpenGWAS data for magnesium included 64,979 Europeans, with GWAS ID ukb-b-7372. The OpenGWAS data for potassium included 64,979 Europeans, with GWAS ID ukb-b-17881. The OpenGWAS data for selenium included 2,603 Europeans, with GWAS ID ieu-a-1077. The OpenGWAS data for vitamin A included 8,863 Europeans, with GWAS ID ukb-b-9596. The OpenGWAS data for vitamin B12 included 64,979 Europeans, with GWAS ID ukb-b-19524. The OpenGWAS data for vitamin B6 included 64,979 Europeans, with GWAS ID ukb-b-7864. The OpenGWAS data for vitamin C included 64,979 Europeans, with GWAS ID ukb-b-19390. The OpenGWAS data for vitamin D included 64,979 Europeans, with GWAS ID ukb-b-18593. The OpenGWAS data for vitamin E included 64,979 Europeans, with GWAS ID ukb-b-6888. The OpenGWAS data for zinc included 2,603 Europeans, with GWAS ID ieu-a-1079. Additionally, the outcome data for AT were obtained from the FinnGen database^2^ under finngen_R10_M13_ACHILLESTEND (DF11–2024.06.24), as shown in Table 1, including 3,434 AT patients and 294,770 normal controls. All participants for both exposure and outcome data were of European ancestry. Detailed information on participants, genetic analysis, imputation, and quality control can be found on the Finngen Biobank website. As the data analyzed in this study were obtained from publicly available databases, ethical approval and informed consent from institutional review boards were not required. These data sources guarantee transparency and reliability, allowing the findings of our study to be shared and discussed across a broader spectrum of medical research.

**Table 1:** Exposure data from the OpenGWAS database: copper, calcium, iron, magnesium, potassium, selenium, zinc, carotene, folate, vitamins A, B6, B12, C, D, and E.

	Trace element	GAWS ID	Sample size
Exposure	Copper	ieu-a-1073	2,603
Exposure	Calcium	ukb-b-8951	64,979
Exposure	Carotene	ukb-b-16202	64,979
Exposure	Folate	ukb-b-11349	64,979
Exposure	Iron	ukb-b-20447	64,979
Exposure	Magnesium	ukb-b-7372	64,979
Exposure	Potassium	ukb-b-17881	64,979
Exposure	Selenium	ieu-a-1077	2,603
Exposure	Vitamin A	ukb-b-9596	8,863
Exposure	Vitamin B12	ukb-b-19524	64,979
Exposure	Vitamin B6	ukb-b-7864	64,979
Exposure	Vitamin C	ukb-b-19390	64,979
Exposure	Vitamin D	ukb-b-18593	64,979
Exposure	Vitamin E	ukb-b-6888	64,979
Exposure	Zinc	ieu-a-1079	2,603
Outcome	Achilles tendon disease	finngen_R10_M13_ACHILLESTEND	3,434

Outcome data from the FinnGen database: Achilles tendon disease.

### 2.3 Instrumental variable processing

According to the STROBE-MR research guidelines, each SNP for micronutrients must go through the following screening steps: First, the genome-wide significance threshold *p <* 5 × 10^−6^ is used. Second, the Clump function is used for linkage disequilibrium (LD) testing, with the standard set at *r*^2^ < 0.001 and kb = 10,000 (24). In addition, the PhenoScanner database is used to exclude SNPs associated with outcomes to eliminate confounders. Finally, the *F*-statistic for each SNP is calculated, and SNPs with *F* < 10 are excluded to avoid bias from weak IVs. At the same time, the following formula is used to calculate the proportion of exposure explained by the instrumental variable (*R*^2^) to quantify the strength of the genetic instrument: *R*^2^ = [2 × Beta^2^ × (1-EAF) × EAF]/[2 × Beta^2^ × (1-EAF) × EAF + 2 × SE^2^ × N×(1-EAF) × EAF], where Beta represents the genetic effect of each SNP, EAF is the effect allele frequency, SE is the standard error, and N is the sample size. To assess the strength of the selected SNPs, the *F*-statistic for each SNP is calculated using the following formula: *F* = *R*^2^(*N*-*k*-1)/*k*(1-*R*^2^), where *R*^2^ represents the proportion of exposure explained by the selected SNP, *N* is the sample size, and *k* is the number of included IVs. SNPs with *F* < 10 are excluded as weak IVs. The remaining independent IVs are used for subsequent MR analysis. MR-STRO is used to detect outliers and adjust for horizontal pleiotropy (25). If horizontal pleiotropy is detected in the IVs, outliers are removed.

### 2.4 Statistical analysis

All analyses in this study were performed using R language (version 4.4.1) and the TwoSampleMR package (26). To conduct a comprehensive and precise study of the causal relationship between micronutrients and AT, we employed various complementary MR methods, using inverse variance weighting (IVW), weighted median, MR-Egger, simple mode, and weighted mode to evaluate potential causal effects (27). Causal effects reflect the impact of a one standard deviation (SD) increase in each input micronutrient on the risk of outcome characteristics, expressed as odds ratios (OR) and their 95% confidence intervals (CI). Sensitivity analyses include tests for heterogeneity, genetic pleiotropy, and leave-one-out analysis. Heterogeneity was tested using the Cochran *Q* test, where *p* > 0.05 indicates no heterogeneity, and *p* < 0.05 suggests the possibility of intergenic heterogeneity. The ideal result of the leave-one-out method is that no significant changes occur after removing each SNP one by one. The MR-Egger method (typically indicated by the intercept of MR-Egger) and the MR-ESTO global test were used to detect horizontal pleiotropy (28, 29). If the results show no pleiotropy and no heterogeneity, the IVW is significant, other methods are significant, and the results are stable. Finally, this study used multivariable MR to analyze the causal relationships between various exposures and diseases, assessing the combined impact and interactions of multiple exposures on disease risk.

## 3 Results

### 3.1 MR analysis

**Figure 3:**
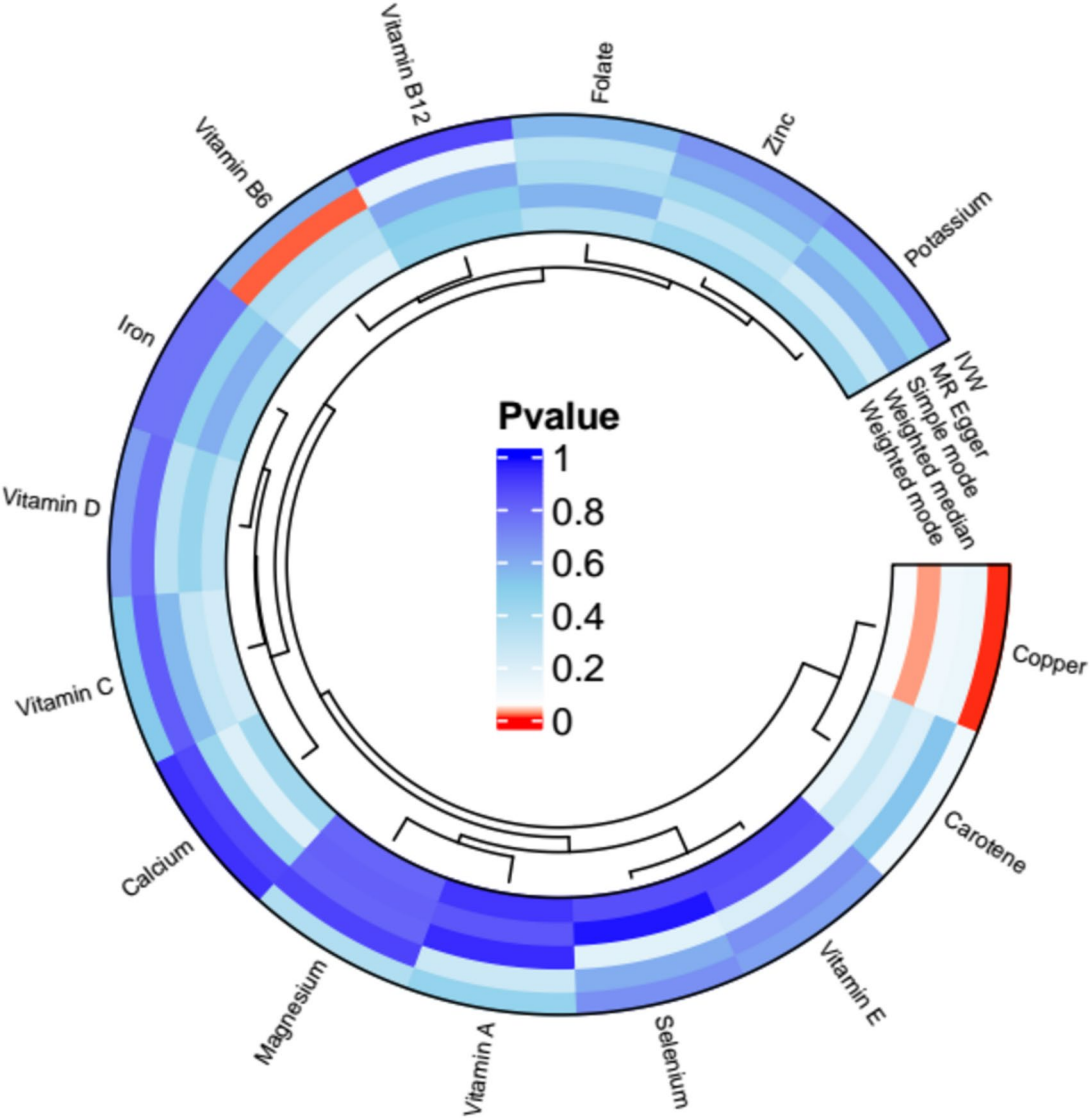
Mendelian randomization (MR) analysis results of exposures (copper, calcium, iron, magnesium, potassium, selenium, zinc, carotene, folate, vitamins A, B6, B12, C, D, and E) and outcome (AT). Five methods: inverse variance weighting (IVW), weighted median, MR-Egger, simple mode, and weighted mode.

Copper, calcium, iron, magnesium, potassium, selenium, zinc, carotene, folic acid, vitamins A, B6, B12, C, D, and E, these 15 micronutrients were treated as exposure factors, and AT as the outcome factor for MR analysis. After multiple corrections, the random effects IVW analysis showed a significant causal relationship between copper (*p* = 0.002, OR = 0.899, 95% CI = 0.839–0.964) and AT. Additionally, the results of the weighted median analysis for copper (*p* = 0.002, OR = 0.892, 95% CI = 0.807–0.89) were consistent with the IVW analysis. However, the MR-Egger method showed: *p* = 0.132, OR = 0.911, 95% CI = 0.827–1.003; the simple mode showed *p* = 0.113, OR = 0.852, 95% CI = 0.724–1.003, with no heterogeneity (*p* > 0.05) and OR > 1. The weighted mode showed *p* = 0.006, OR = 0.891, 95% CI = 0.809–0.981, with no heterogeneity (*p* > 0.05) and OR < 1. MR result analysis as shown in Figure 3 and Supplementary File 1.

**Figure 4:**
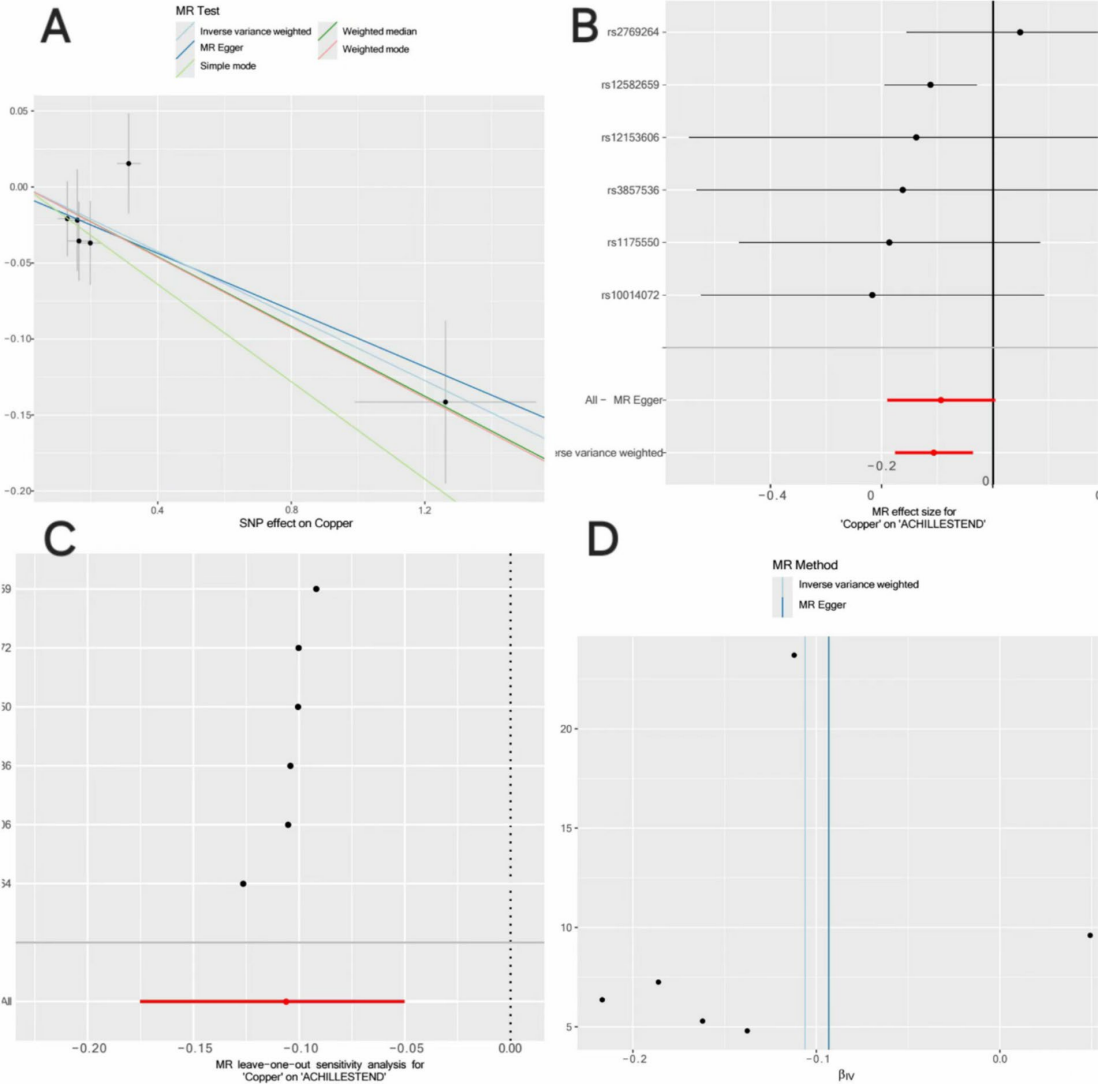
Mendelian randomization analysis of the causal relationship between copper and AT. (A) Scatterplots for the causal association between copper and AT. The slope of a straight line indicates the magnitude of causality. Black dots represent genetic instruments included in the main Mendelian randomization analysis. (B) Forest map visualization of the causal impact of each SNP on AT risk. (C) “Leave-one-out” plots for the causal association between copper on AT risk. (D) Funnel plot showing heterogeneity of SNP.

Disease-related micronutrients were screened using the R package. The analysis revealed that copper’s pleiotropy had *p* = 0.727 (*p* > 0.05), showing an association with AT (Supplementary File 2). MR analysis was conducted on copper, a micronutrient associated with AT (Supplementary File 3), and the random-effects IVW analysis indicated copper (*p* = 0.003, OR = 0.899, 95% CI = 0.839–0.964). Pleiotropy tests (Supplementary File 4) and heterogeneity tests (Supplementary File 5) were also conducted, with *p-*values all greater than 0. Outlier detection results indicated that all combined *p*-values for outlier detection were >0.05 (Supplementary File 6), and no SNP outliers were detected in individual SNP outlier testing (Supplementary File 7). Additionally, through a scatter plot, we observed that the results of SNPs on exposure and outcome factors were consistent across the five methods used. Leave-one-out sensitivity analysis showed that removing individual SNPs did not excessively impact the MR analysis. The funnel plot also showed a symmetric distribution in Figure 4.

### 3.2 Multivariable MR analysis

**Figure 5:**
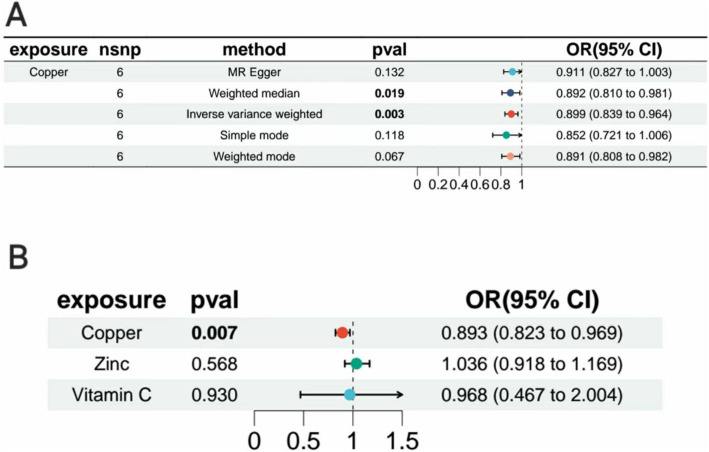
Forest plot. (A) Forest plot of Mendelian randomization analysis of copper with inverse variance weighting (IVW), weighted median, MR-Egger, simple mode, and weighted mode. (B) Forest plots for inverse variance-weighted Mendelian randomization analysis of zinc, vitamin C, and copper.

Zinc and vitamin C, which are related to AT among the 15 micronutrients, were selected for multivariable MR (Supplementary File 8) (30, 31). The results showed that zinc (*p* = 0.568, OR = 1.036, 95% CI = 0.918–1.169) and vitamin C (*p* = 0.962, OR = 0.968, 95% CI = 0.467–2.004) did not have independent causal effects on AT. Copper (*p* = 0.007, OR = 0.893, 95% CI: 0.823–0.969) had an independent causal effect on AT, and copper is a protective factor for AT. Heterogeneity and pleiotropy tests were conducted, yielding *Q-*values with *p* > 0.05, indicating no significant heterogeneity or pleiotropy (Supplementary File 9). We compared the forest plot of copper with the forest plots of copper, zinc, and selenium, showing the causal relationship between copper and AT in Figure 5. Thus, it can be concluded that copper may be a protective factor for AT.

## 4 Discussion

The AT is a complex musculoskeletal disorder, essentially characterized by a failure in the healing response of the Achilles tendon at the heel. Patients with AT often experience pain, swelling, and dysfunction around the Achilles tendon, significantly impacting their quality of life, which is closely related to micronutrients (13–20). This study used MR to investigate the causal relationships between 15 micronutrients (copper, calcium, iron, magnesium, potassium, selenium, zinc, carotenoids, folic acid, vitamin A, vitamin B6, vitamin B12, vitamin C, vitamin D, and vitamin E) and AT. MR analysis showed that copper had a causal relationship with AT and that copper is a protective factor for AT.

Copper is an essential micronutrient in the human body, and as an important trace metal, it is required for the catalysis of many critical cellular enzymes (32). Lysyl oxidase (LOX) is implicated in the formation of collagen fiber crosslinks, and copper is a cofactor for LOX, and therefore may influence the differentiation of mesenchymal stem cells to tendon cells (33). A correlation between LOX activity and dietary copper intake has been noted (34). Previous studies have demonstrated that copper ions can drive inflammation via the mitochondrial signaling pathway, which can also regulate the epigenetic state of immune cells (35, 36). The role of copper in regulating inflammation has gained increasing attention, making it crucial to clarify the relationship between copper and Achilles tendinopathy.

The human body primarily relies on dietary intake to maintain adequate copper levels, which are vital for both physiological and psychological health (37, 38). Copper is involved in numerous cellular processes, including hematopoiesis, immune defense, and free radical scavenging (39). Nonetheless, an excess of copper in the body is linked to several diseases, including Alzheimer’s disease and Wilson’s disease, which are neurodegenerative conditions (40). The adult human body contains approximately 100 mg of copper, with over half distributed in the bones and muscles. The highest concentrations are found in the liver, kidneys, and brain, followed by the heart and hair. Only about 5% of copper is present in the blood, with approximately 95% bound to ceruloplasmin (CP) and the remainder bound to albumin and amino acids (41, 42). Copper plays a significant role in the body’s anti-inflammatory processes. Recent studies suggest that copper can be utilized as an adjunct in the treatment of osteoarthritis and cancer (43, 44).

Copper deficiency can lead to neutropenia, anemia, osteoporosis, and cause muscle cramps and abnormal muscle tone (45). The human body requires copper to catalyze many important cellular enzymes (32, 46). Copper can regulate inflammatory responses by modulating the functions of T helper cells, B cells, neutrophils, natural killer cells, and macrophages (47, 48). It can also induce the activity of LOX and regulate the synthesis of prostaglandins (49–51). It induces or mimics superoxide dismutase activity, reduces the permeability of synovial lysosomes, and regulates histamine production (52). Therapeutic interventions involving copper can prevent or reduce the deterioration of congested areas and wound size after burns (53). Nano-copper, when applied locally to burn areas, has significant anti-inflammatory effects by reducing cytokine expression (54). ROS are key signaling molecules in inflammation; oxidative stress occurs when ROS accumulate beyond a cell’s antioxidant capacity, leading to oxidative damage. Oxidative stress is associated with various inflammatory diseases, and studies have shown that ultra-small copper nanoparticles at very low doses can protect cells from ROS damage and significantly promote healing (55–57). However, excess copper in cells generates free radicals and increases oxidative stress (40). Most studies have shown that both copper deficiency and excess are harmful to the human body (32, 58). Therefore, copper homeostasis is causally related to the progression of AT. The U.S. Institute of Medicine’s Food and Nutrition Board recommends a daily copper intake of about 700 micrograms for adults (59). Copper sulfate can promote tendon regeneration by enhancing the recruitment of mesenchymal stromal cells (MSC) to the site of injury. These cells secrete growth factors and other substances, counteract oxidative stress, and stabilize collagen fibers, thereby accelerating tendon healing (60). New research shows that ultra-small copper-based enzyme clusters can improve Achilles tendinopathy by inhibiting acute oxidative stress (61). Obesity is a risk factor for AT (62), Copper inhibits phosphodiesterase 3 (PDE3) during the breakdown of fat cells for energy, and PDE3 hydrolyzes cyclic AMP (cAMP), which promotes fat breakdown (63). Therefore, copper is a protective factor against AT.

Our study provided new insights into the treatment of AT, but there were also some limitations. First, to reduce the interference of population stratification, we only performed stratified analyses based on European ancestry, without considering factors such as age, dietary habits, and lifestyle, which may introduce bias and fail to fully reflect the actual situation of broader populations. Moreover, in genome-wide association studies, we used the conventional significance level *p*-value = 5 × 10^−8^, but we did not obtain enough SNPs for MR analysis. Therefore, we relaxed the significance level to 5 × 10^−6^ to obtain more candidate SNPs, though this may increase the false positive rate and affect the reliability of the results. Future studies need to optimize the design, include more diverse population characteristics, and adopt stricter statistical standards to ensure the robustness and generalizability of the results.

## 5 Conclusion

In conclusion, our study clarified the causal relationship between copper and AT through MR analysis. This insight not only offers a new perspective for subsequent mechanistic studies, but also provides new scientific evidence and strategies for the clinical application of copper in the prevention and treatment of AT. Through in-depth analysis, we found that copper plays a key role in the occurrence and progression of AT, pointing the way for future research. Furthermore, this discovery provides an important reference for clinicians when formulating treatment plans, potentially improving patient prognosis and quality of life.

## Data Availability

The original contributions presented in the study are included in the article/Supplementary material, further inquiries can be directed to the corresponding author.

## Footnotes

^1^https://gwas.mrcieu.ac.uk/

^2^https://www.finngen.fi/
